# Advances in machine learning-enhanced nanozymes

**DOI:** 10.3389/fchem.2024.1483986

**Published:** 2024-10-17

**Authors:** Yeong-Seo Park, Byeong Uk Park, Hee-Jae Jeon

**Affiliations:** ^1^ Department of Advanced Mechanical Engineering, Kangwon National University, Chuncheon, Republic of Korea; ^2^ Department of Mechanical and Biomedical Engineering, Kangwon National University, Chuncheon, Republic of Korea

**Keywords:** nanozyme, machine learning, bioapplication, colorimetric, biosensing

## Abstract

Nanozymes, synthetic nanomaterials that mimic the catalytic functions of natural enzymes, have emerged as transformative technologies for biosensing, diagnostics, and environmental monitoring. Since their introduction, nanozymes have rapidly evolved with significant advancements in their design and applications, particularly through the integration of machine learning (ML). Machine learning (ML) has optimized nanozyme efficiency by predicting ideal size, shape, and surface chemistry, reducing experimental time and resources. This review explores the rapid advancements in nanozyme technology, highlighting the role of ML in improving performance across various bioapplications, including real-time monitoring and the development of chemiluminescent, electrochemical and colorimetric sensors. We discuss the evolution of different types of nanozymes, their catalytic mechanisms, and the impact of ML on their property optimization. Furthermore, this review addresses challenges related to data quality, scalability, and standardization, while highlighting future directions for ML-driven nanozyme development. By examining recent innovations, this review highlights the potential of combining nanozymes with ML to drive the development of next-generation diagnostic and detection technologies.

## 1 Introduction

Nanozymes, a class of nanomaterials that mimic the catalytic activities of natural enzymes, have revolutionized various scientific fields since their introduction (Gao and Yan, 2016; Wu et al., 2019). The concept of nanozymes was first coined in 2004, marking the advent of a new frontier in nanotechnology (Wang et al., 2020; Wu et al., 2021a; Salvador-Morales and Grodzinski, 2022). Subsequently, the field has witnessed significant milestones such as the development of gold nanozymes in 2008, which opened new avenues for mimicking enzyme functions (Wang et al., 2020; Liang and Yan, 2019). Further, advancements in metal oxide nanozymes were recorded around 2010, demonstrating applications in remediation and biosensors. Carbon-based nanozymes emerged in 2012 owing to their high catalytic efficiency (Wu et al., 2019; Wong et al., 2021). Such developments illustrate the dynamic evolution of nanozyme technology and highlight its growing importance in various applications. Due to their diverse catalytic properties, nanozymes have become invaluable tools for developing biosensors and diagnostic applications.

Among the different types of nanozymes, electrochemical nanozymes are known for their ability to induce or enhance electrical signals, making them essential for biosensing applications requiring high sensitivity and precision, including the detection of trace amounts of biomarkers in bodily fluids. Such nanozymes enable the development of highly sensitive biosensors suitable for point-of-care diagnostics and real-time monitoring (Geng et al., 2022; Sharifi et al., 2020). Alongside other types of nanozymes, chemiluminescent nanozymes offer exceptional specificity and sensitivity, often down to a single-molecule level, making them ideal for environments where minimal background interference is crucial, such as in complex biological matrices or *in vivo* imaging (Wu and Qu, 2015; Roda et al., 2016).

Commercial biosensors based on nanozyme technologies possess practical potential for real-world bioapplications (Yang et al., 2015). With advancements in nanozyme technology, biosensors have increasingly leveraged the unique properties of colorimetric, electrochemical, and chemiluminescent nanozymes for detecting a wide range of biological targets, from pathogens to biomolecules, with high sensitivity, specificity, and versatility (Wang et al., 2024; Wu et al., 2021b; Chen X. et al., 2022). Such a multi-modal approach, combining visual, electrical, and luminescent signals, ensures the suitability of nanozyme-based biosensors to diverse applications, ranging from point-of-care diagnostics to environmental monitoring (Liu X. et al., 2021; Saleh and Hassan, 2023).

The advent of artificial intelligence (AI) has revolutionized various fields, and integrating machine learning (ML) into nanozyme-based bioapplications presents a significant leap forward in the field (Mujtaba et al., 2021; Yoon et al., 2024a). ML has the ability to process massive amounts of data and classify complex patterns, which has been instrumental in enhancing the functionality and applications of nanozymes in biotechnology. However, despite these advancements, nanozyme development still faces several challenges, including the need to optimize catalytic efficiency, stability, and specificity for various bioapplications. ML addresses these challenges by predicting optimal nanozyme properties, reducing experimental time and resource consumption, and enabling more precise tuning of their catalytic activities. The initial steps towards their integration began in the early 2010s, with predictive modeling used to better understand and optimize nanozyme properties (Butler et al., 2018). By 2020, ML became a key component in the real-time monitoring and analysis of nanozyme activity, allowing highly precise and dynamic bioapplications (Mou et al., 2022). The convergence of ML and nanozyme technology has led to the development of smart biosensors and diagnostic tools that can adapt and respond to changing conditions in real time, greatly enhancing their utility in medical diagnostics, environmental monitoring, and other bioapplications (Pramanik et al., 2020; Weerathunge et al., 2019; Xu L. et al., 2022), including the development of more sophisticated diagnostic tools that provide real-time feedback, adaption to complex biological environments, and personalized medicine applications of ML-driven nanozymes tailored to individual patient requirements (Cui et al., 2020). The synergy between the two cutting-edge technologies holds great promise for the future of biotechnology.

**FIGURE 1:**
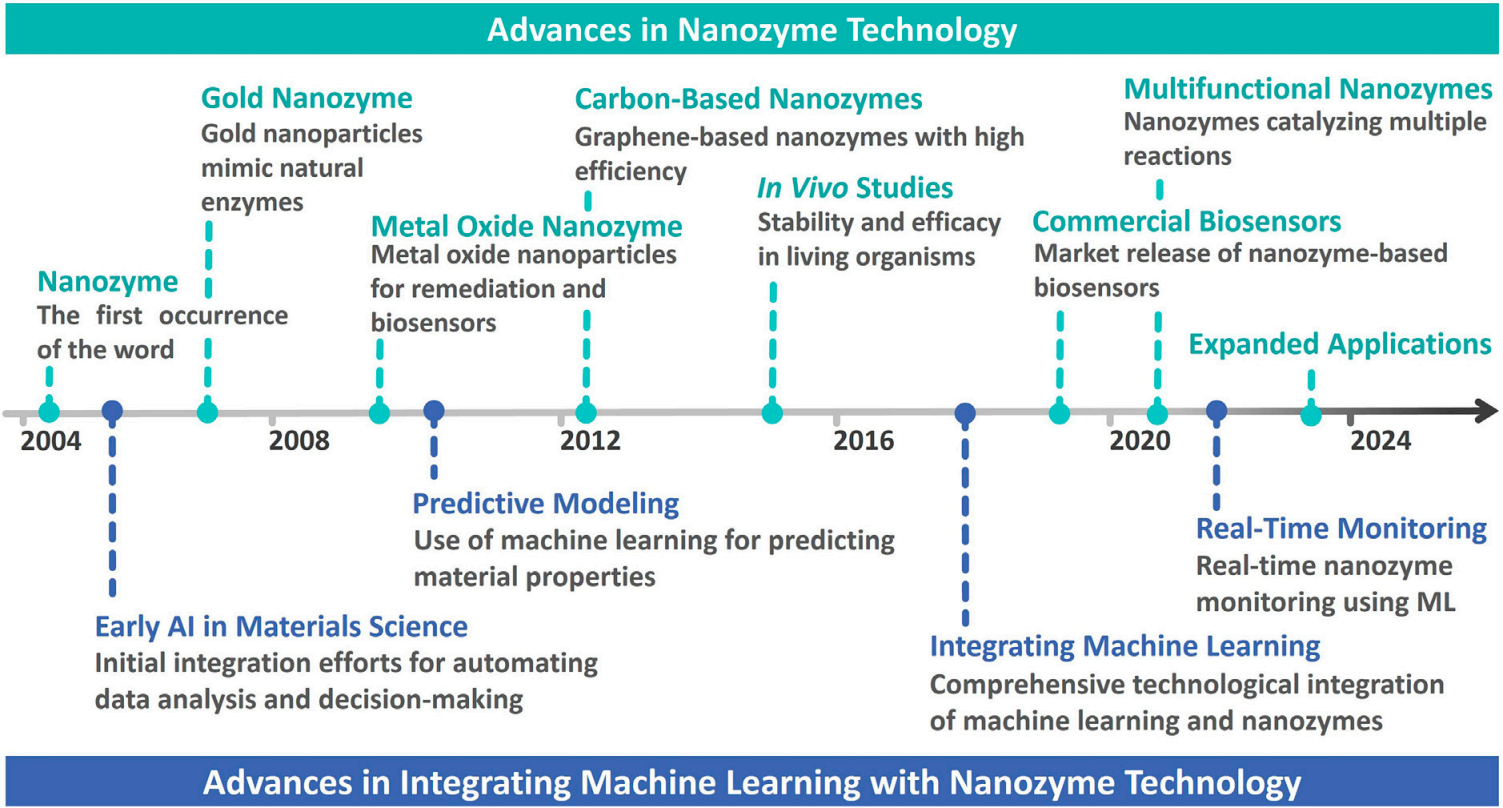
Brief timeline of advancements in integrating machine learning with nanozyme technology.

This article aims to review developments in nanozymes, their bioapplications, and their integration with ML, as illustrated in Figure 1. Since the inception of nanozymes in 2004, significant milestones have been achieved, including the development of gold nanozymes in 2008, pioneering the mimicry of natural enzymes, and further advancements including metal oxide and carbon-based nanozymes in 2010 and 2012, respectively (Manea et al., 2004; Li et al., 2008; Wei and Wang, 2013; Sun et al., 2018). Such developments have significantly promoted nanozyme applications, particularly in biosensing and diagnostics. This study highlights the increasing integration of ML with nanozyme-based bioapplications, a trend that began in the early 2010s with predictive modeling, and has since evolved into a critical component of real-time monitoring and dynamic applications in biotechnology (Noll and Henkel, 2020; Wagner and Rondinelli, 2016). With evolution in the synergy between nanozymes and ML, further innovations are expected, leading to more sophisticated, responsive, and personalized diagnostic tools that can adapt to complex biological environments.

## 2 Nanozyme development and catalytic mechanisms

### 2.1 Types of nanozymes

**FIGURE 2:**
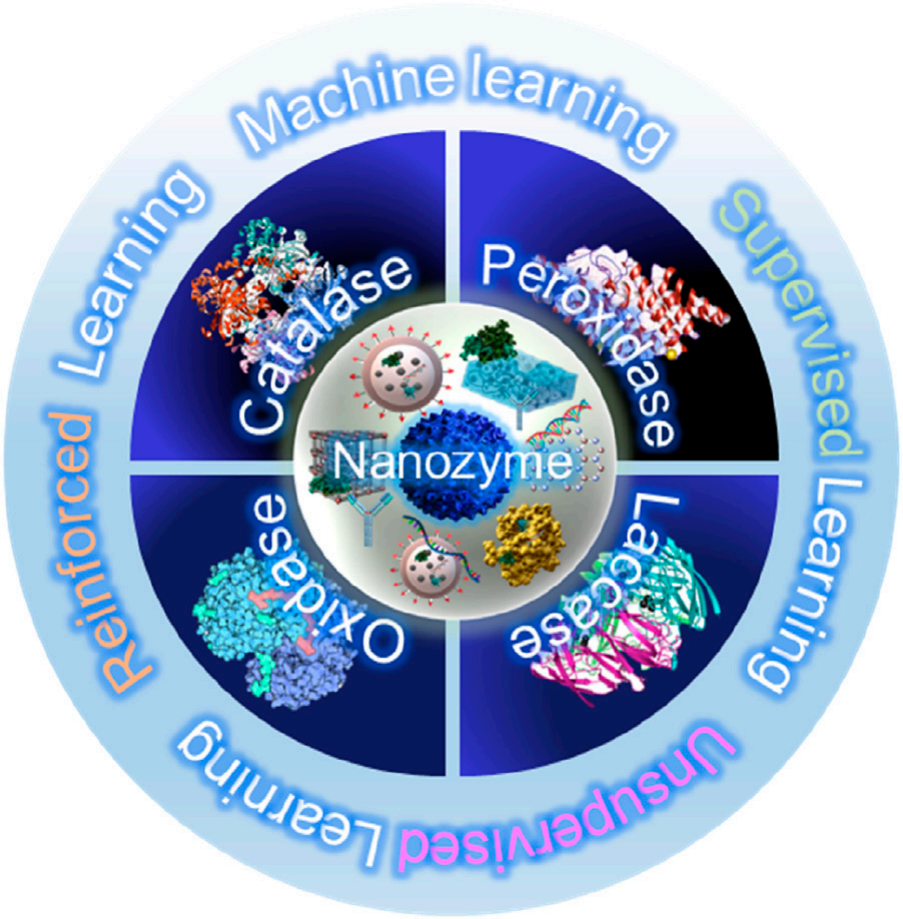
Types of nanozymes and their enzymatic functions.

Nanozymes are engineered nanomaterials that emulate the catalytic functions of natural enzymes, as shown in Figure 2. They can be broadly classified into several types based on their material composition (Huang et al., 2019). Metallic nanozymes, including gold, silver, and platinum, are well known for their high catalytic efficiency and stability (Lou-Franco et al., 2021). Metallic nanozymes often exhibit strong enzyme-like activities owing to their ability to easily donate or accept electrons during redox reactions, which is crucial for mimicking enzymes such as oxidases, peroxidases, and catalases. Such nanozymes are widely used in biosensing and diagnostic applications because of their reliable and stable catalytic properties (Wang et al., 2018). Metal oxide nanozymes, including those based on iron, cerium, and manganese oxides, are another key category (Liu Q. et al., 2021). Particularly, such nanozymes are robust, multi-functional, and possess the ability to simultaneously perform multiple types of catalytic reactions. For example, cerium oxide nanozymes can switch between different oxidation states, enabling them to mimic both catalase and superoxide dismutase activities. Their robustness and multi-functionality make them suitable for environmental applications such as pollutant degradation and biosensor development (Meng et al., 2020). Carbon-based nanozymes, including graphene, carbon nanotubes, and carbon dots, represent a rapidly expanding category owing to their high surface area, conductivity, and tunable catalytic properties (Yang et al., 2020). Such nanozymes offer numerous active sites and are easy to chemically modify for enhancing their catalytic properties. Moreover, their excellent conductivity aids electron transfer processes, which are essential for mimicking peroxidase activity. Consequently, carbon-based nanozymes are being increasingly used in biosensors, environmental remediation, and energy-related applications (Li S. et al., 2019).

### 2.2 Catalytic mechanisms and factors affecting catalytic activity

The catalytic mechanisms of nanozymes are influenced by several nanoscale properties crucial to their functionality (Huang et al., 2019). Size and shape are critical factors to determine the surface area available for catalytic reactions and active site distributions (An and Somorjai, 2012). Nanozymes of smaller sizes typically have a higher surface-area-to-volume ratio, which enhances their interaction with substrates, resulting in higher catalytic efficiency (Wang et al., 2019). The composition of nanozymes, including specific metals or metal oxides used, dictates the type of catalytic activity. Different materials offer various enzyme-mimicking functions (Das et al., 2021). For instance, platinum-based nanozymes are highly effective in hydrogenation reactions, whereas gold-based nanozymes exhibit excellent oxidase-like activity. The intrinsic properties of such materials allow effective replication of specific enzymatic functions. Additionally, surface chemistry plays a significant role in catalytic performance, as the presence of functional groups or surface modifications improve substrate binding and provide protection against degradation (Jing et al., 2013). Surface modifications can be tailored to improve the interactions between nanozymes and their target substrate, thereby enhancing specificity and catalytic turnover. For example, attaching specific ligands to the surface of a nanozyme can help in selectively binding certain biomolecules, mimicking the specificity of natural enzymes (Soares et al., 2021). The aforementioned factors influence the stability and reactivity of nanozymes. For example, a change in pH can alter the charge on the nanozyme surfaces, affecting their interaction with substrates. Similarly, temperature variation can affect the kinetic energy of a system, thereby affecting the catalytic reaction rate.

### 2.3 Methods of nanozyme development and limitations

Traditional nanozyme designs rely on empirical synthesis methods including chemical reduction, sol-gel processes, and hydrothermal synthesis (Chadha et al., 2022). However, conventional methods often lack the precision required to control nanoparticle size, shape, and composition. Consequently, the catalytic properties of the synthesized nanozymes vary widely, making it challenging to achieve consistent performance (Sun et al., 2018; Singh, 2019). The empirical nature of such approaches makes them time-consuming and resource-intensive, requiring extensive experimentation to identify optimal synthesis conditions and functional properties. The trial-and-error approach often leads to inefficiencies, as researchers are required to test multiple variables including reaction time, temperature, and precursor concentration to fine-tune nanozyme properties. Moreover, traditional methods often struggle to achieve high specificity and stability because nanozymes typically do not match the substrate specificity of natural enzymes and can suffer from aggregation, oxidation, or loss of activity over time.

To overcome such limitations, recent advancements have focused on integrating computational approaches and ML into design processes (Gupta et al., 2021). Modern strategies enable more precise prediction of nanozyme properties by utilizing large datasets to model and predict the effects of different synthesis parameters on nanozyme performance. By leveraging data-driven models, researchers can optimize the nanozyme characteristics more efficiently, leading to the development of effective and stable nanozymes with enhanced catalytic performance for a wide range of bioapplications. New approaches not only accelerate the design process but also improve the reproducibility and scalability of nanozyme production.

## 3 Integration of ML in nanozyme development

### 3.1 Overview of ML techniques

**FIGURE 3:**
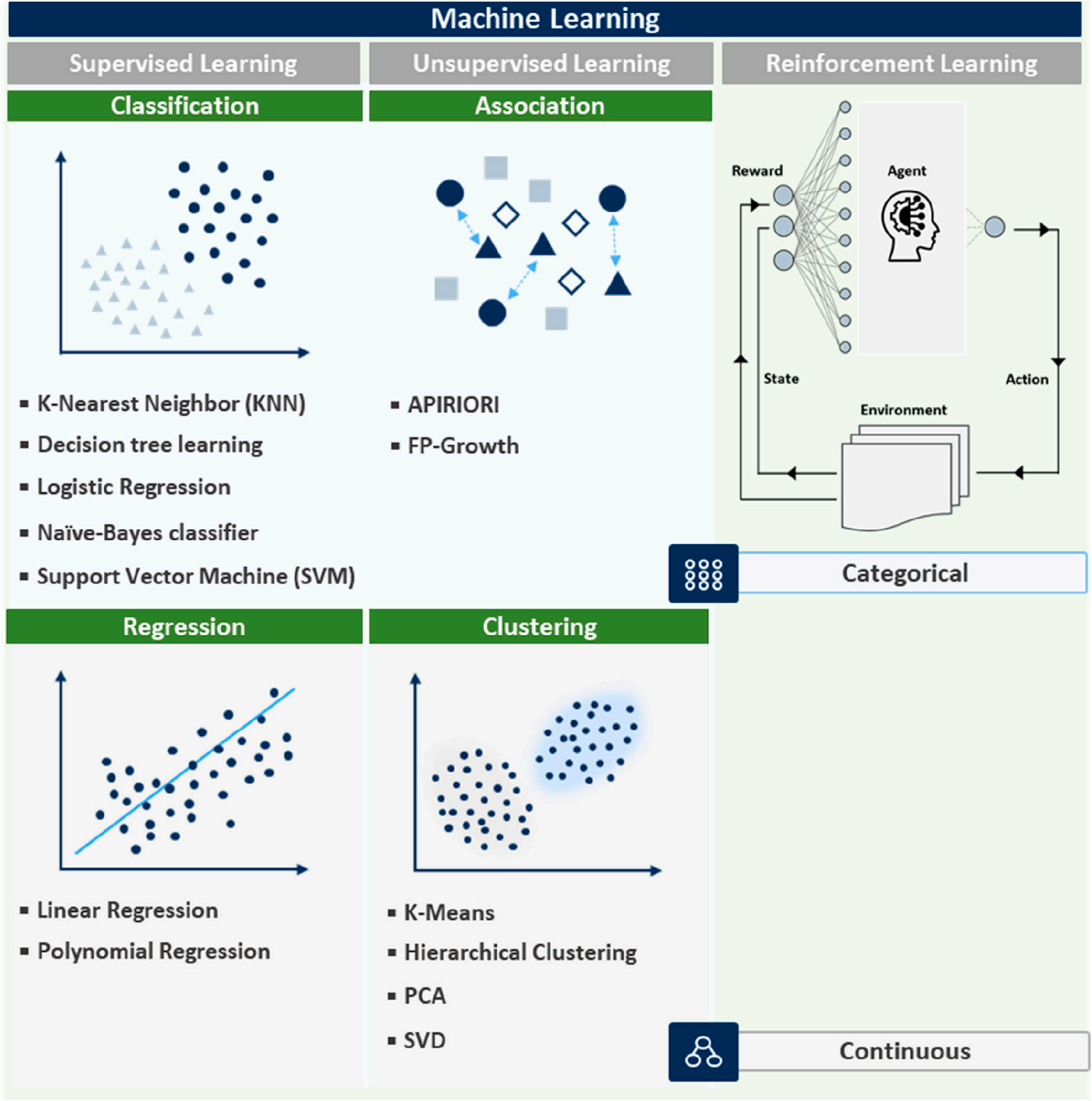
Overview of ML techniques: supervised, unsupervised, and reinforcement learning with data type distinctions.

Recently, ML is being increasingly integrated into nanozyme design, offering powerful tools for predicting and optimizing nanozyme properties (Chen et al., 2023). The integration of ML into nanozyme research has revolutionized the field, enabling more precise control of the design process and significant time reduction for experimentation. By analyzing vast datasets, ML models uncover complex relationships between nanozyme characteristics and catalytic performances, which can be difficult to discern using traditional methods. Different ML techniques such as supervised, unsupervised, and reinforcement learning are applied based on specific nanozyme or sensor requirements, as shown in Figure 3. The effectiveness of these techniques is closely tied to the methods used for signal collection (Chadha et al., 2022). Signal collection plays a critical role in obtaining high-quality data, which is essential for accurate ML predictions. For example, colorimetric sensors collect optical signals based on visible color changes, electrochemical sensors measure electrical signals, and chemiluminescent sensors collect light emission signals. The nature of the signal collected directly impacts how the data is processed and the ML model applied (Gupta et al., 2021). The application of machine learning techniques in nanozyme-based sensors is highly dependent on the programming code used to implement these techniques. Each ML method—supervised, unsupervised, and reinforcement learning—requires a distinct coding approach that impacts how sensor data is processed and how samples are detected. For example, in supervised learning, the programming code is primarily focused on training the model using labeled datasets (Chen et al., 2023). The code typically includes steps for loading data, preprocessing it (e.g., normalizing or encoding features), training the model, and making predictions. In the case of colorimetric sensors, this involves predicting the color change based on input features such as concentration levels, with code that handles both the training and evaluation phases efficiently.

Supervised learning uses labeled data and is particularly effective for regression and classification tasks, predicting outcomes such as catalytic efficiency, stability, and specificity of nanozymes (Li Y. et al., 2023). Considering nanozyme design, supervised learning algorithms can be trained on experimental data to predict the effects of changes in synthesis conditions (including temperature, pH, and reactant concentration) on the final nanozyme properties (Li Y. et al., 2023; Zhuang et al., 2024). Such a predictive capability enables researchers to fine-tune nanozyme characteristics before physical synthesis, saving time and resources. Supervised learning is especially useful for applications such as colorimetric sensors, where precise detection and labeled data are readily available. In contrast, unsupervised learning relies on code that can discover patterns in unlabeled data, such as clustering or association rule mining. For electrochemical sensors, the code is used to analyze complex electrical signal data and group samples based on their signal characteristics (Sun et al., 2018). This involves designing algorithms that can process raw signals and cluster them into meaningful groups without prior knowledge of the data structure.

Unsupervised learning, including clustering and association techniques, identifies patterns within unlabeled data to discover new nanozyme classes and understand their properties (Arya et al., 2023; Ghahramani, 2003; Barlow, 1989). For instance, clustering algorithms group nanozymes based on their catalytic behaviors or structural features, revealing previously unrecognized relationships that can lead to the development of novel nanozyme types. However, association techniques can identify common features among high-performance nanozymes and guide the synthesis of new variants with enhanced functions. Unsupervised learning is particularly effective for electrochemical sensors, where hidden patterns in complex signal data can be uncovered and used to optimize sensor performance.

Reinforcement learning requires code that facilitates interaction between the model and its environment. In chemiluminescent sensors, the code must simulate different sensor conditions and adjust sensor parameters in real-time based on feedback from the environment. The core of the reinforcement learning code involves setting up the environment, defining a reward function, and iterating through learning episodes to optimize performance. Reinforcement learning optimizes processes by learning from interactions, which is useful for refining synthesis conditions to enhance nanozyme performance (Chen et al., 2023). During nanozyme development, reinforcement learning can be applied to iteratively improve synthesis protocols. By simulating different synthesis scenarios and learning from obtained outcomes, reinforcement learning algorithms can recommend optimal pathways for producing nanozymes with the desired properties. The approach is particularly valuable in dynamic and complex systems where the best synthesis strategy may not be apparent from initial conditions. Reinforcement learning is highly suitable for chemiluminescent sensors, which require real-time performance optimization, especially in fluctuating environments like *in vivo* diagnostics or environmental monitoring.

The aforementioned methods are visually categorized in Figure 3, illustrating their applications in handling both continuous and categorical data and their relevance in nanozyme research. For example, supervised learning works best for predicting quantitative outcomes (continuous data), such as catalytic rates, while unsupervised learning helps classify nanozymes into functionality-based categories (categorical data). The choice of ML technique and signal collection method are both essential for ensuring accurate predictions, optimized nanozyme performance, and reliable results across various bioapplications. By effectively utilizing ML techniques, researchers can accelerate the discovery and optimization of nanozymes, leading to more efficient and sustainable solutions in various applications, including biomedicine, environmental remediation, and industrial catalysis (Ahmed et al., 2022; Ngwabebhoh and Yildiz, 2019).

### 3.2 Application of ML in predicting nanozyme properties

ML is crucial for predicting key nanozyme characteristics including catalytic activity, specificity, and environmental stability. By processing large datasets, ML models can identify the most influential factors affecting nanozyme performance such as particle size, shape, and surface chemistry (Wei et al., 2022). The ability to analyze complex and large-scale data allows researchers to determine attributes that most significantly affect the efficiency and functionality of nanozymes, providing valuable insights that guide the design and optimization processes. For example, regression models can predict the optimal conditions for catalytic reactions, including ideal temperature, pH, and reactant concentration, to achieve maximum efficiency (Flynn and Chang, 2024). Such models are essential for narrowing down the vast array of potential experimental conditions to the most promising ones, thereby saving time and reducing costs associated with trial-and-error approaches. Conversely, classification models sort nanozymes based on their functional categories, such as oxidase-like, peroxidase-like, or catalase-like activities. Sorting helps to quickly identify appropriate nanozymes for specific applications such as biosensing or pollutant degradation (Li X. et al., 2019; Li X. et al., 2023).

Clustering techniques group nanozymes with similar properties, thereby facilitating the discovery of new variants with enhanced capabilities (Ai et al., 2022). For instance, clustering can reveal subgroups of nanozymes that share unique catalytic properties, which may not be immediately apparent through conventional analysis. By studying the clusters, researchers can identify common features that contribute to high performance and use the knowledge to design new nanozymes with improved functionalities. The predictive power accelerates process development, enabling efficient and better suited nanozyme designs for specific applications. Ultimately, the integration of ML into nanozyme research not only accelerates the discovery of new nanozyme variants but also enhances their performance in real-world applications, ranging from environmental remediation to advanced medical diagnostics. By leveraging the power of ML, researchers can push the boundaries of nanozymes for obtaining innovative solutions in various fields.

### 3.3 Case studies of ML-assisted nanozyme development

Several case studies have highlighted the effectiveness of ML in improving nanozyme designs. For instance, supervised learning has been used to enhance the catalytic activity of metal oxide nanozymes by predicting the influence of factors, such as size and surface area (Xu D. et al., 2022). In such studies, ML models have successfully identified optimal nanoparticle dimensions and surface characteristics that maximize catalytic efficiency, allowing for the fine-tuning of nanozyme properties to meet specific functional requirements. Another study has applied clustering and regression models to optimize the stability of gold nanozymes under varying pH and temperature conditions, resulting in more robust biosensors (Sun et al., 2021). Such ML-driven approaches have been particularly effective in identifying the precise environmental conditions that gold nanozymes can withstand, ensuring their stability and prolonged activity under challenging conditions including those found in biological or environmental samples. Thus, directly contributions have led to the development of reliable and durable biosensors.

Additionally, reinforcement learning has been employed to fine-tune synthesis parameters for carbon-based nanozymes to achieve optimal performance (Lewandowska et al., 2021). Particularly, reinforcement learning models have been adept at iteratively adjusting synthesis variables such as reaction time, temperature, and precursor concentrations, learning from each outcome to progressively improve nanozyme performance (Kulkarni et al., 2022). Thus, significant enhancements in the catalytic capabilities and stabilities of carbon-based nanozymes have been recorded, making them more effective for environmental remediation and energy conversion applications. The examples depicted in Figure 3 demonstrate the creation of more efficient, stable, and application-specific nanozymes using ML-based approaches. By integrating ML into the design process, researchers can precisely control nanozyme properties and tailor them to meet the specific requirements of diverse applications. The success of the aforementioned case studies underscores the transformative impact of ML on nanozyme research, offering a powerful tool for innovation in this field (Zheng et al., 2024; Cao et al., 2023).

Incorporating ML into nanozyme design represents a significant advancement, enabling the development of more targeted and effective nanozymes for use in diverse fields, such as biotechnology, medicine, and environmental science. As ML techniques continue to evolve, their applications in nanozyme research are expected to expand, driving further innovation and specialization in this rapidly growing field. The ongoing development of ML algorithms, coupled with increasing computational power, is expected to likely produce more sophisticated and efficient nanozyme designs, paving the way for groundbreaking advancements across multiple scientific and industrial domains.

## 4 Recent advances in nanozyme with ML application

### 4.1 Colorimetric sensors and ML applications

**FIGURE 4:**
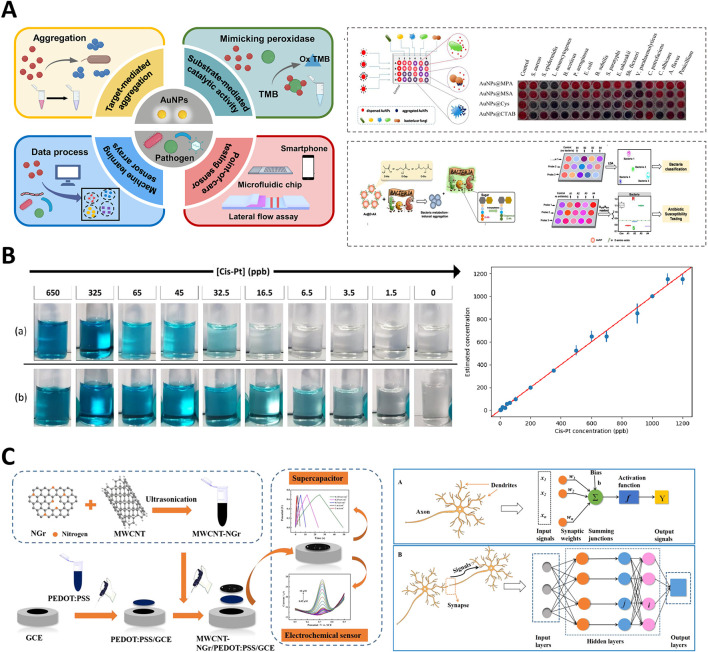
Integration of gold nanoparticles and carbon nanotube-based nanozymes in colorimetric and electrochemical sensors. **(A)** Schematic of gold nanoparticle-based colorimetric pathogen detection systems. Adapted with permission (Yang et al., 2022) copyright 2022, MDPI. **(B)** Colorimetric detection of cisplatin at ppb levels using nanocatalyst-enhanced assays. Adapted with permission (Mastronardi et al., 2022), copyright 2022, MDPI. **(C)** Multi-walled carbon nanotube-N-doped graphene nanohybrid for electrochemical sensing and energy storage applications. Adapted with permission (Xue et al., 2020), copyright 2020, American Chemical Society.

Recently, significant progresses have been made in the development of nanozyme-based colorimetric sensors, particularly through the integration of ML techniques. The innovations have expanded the capabilities of biosensing, diagnostics, and environmental monitoring, highlighting the potential of combining nanotechnology with computational approaches. The application of ML to sensors has enhanced their ability to process complex colorimetric data, such as RGB values, thereby enabling more precise detection and quantification of analytes in various environments. For instance, in studies involving the detection of cisplatin (Cis-Pt) at parts per billion (ppb) levels, as shown in Figure 4A, colorimetric changes have been accurately measured and correlated with cis-Pt concentration, demonstrating the sensitivity of the system (Yang et al., 2022). Table 1 provides an overview of various nanozyme-based colorimetric reactions, summarizing their associated colorimetric reactions, color spaces utilized, ML methods applied, and corresponding limits of detection (LOD). The concise compilation highlights the integration of ML techniques for enhancing the sensitivity and specificity of nanozyme-based diagnostic and monitoring applications in different fields.

**TABLE 1:** Overview of nanozyme-based colorimetric reactions integrated with machine learning for advanced diagnostic and monitoring applications.

Nanozyme type	Colorimetric reaction	Color space	Application	Machine learning method	LOD	Ref.
PtNPs	Oxidation Reaction	RGB	Point-of-care (POC) testing	K-Nearest Neighbors (KNN) Dynamic Time Warping (DTW)	0.0154 μM	Mastronardi et al. (2022)
Fe-N-C single-atom nanozyme (SAN)	Oxidation Reaction	RGB	Point-of-care (POC) testing, Environmental monitoring	Linear Discriminant Analysis (LDA), Hierarchical Clustering Analysis (HCA)	0.5 μM	Shen et al. (2022)
h-PB NPs	Oxidation Reaction	RGB	Point-of-care (POC) testing	Artificial Neural Network (ANN)	0.0126 μM	Yu et al. (2022)
Ni/CoMoO4	Oxidation Reaction	RGB, HSV	Clinical diagnostics, Environmental monitoring	Support Vector Machine (SVM)	0.33 μM	Dang et al. (2022)
Metal-nanoparticle-supported nanozymes (MNNs)	Oxidation Reaction	RGB	Clinical diagnostics	Principal Component Analysis (PCA), Hierarchical Clustering Analysis (HCA)	−	Lu et al. (2022a)
Fe_3_O_4_ NPs	Oxidation Reaction	RGB	Clinical diagnostics, Forensic investigations	Multi-channel convolutional neural network (MC-CNN)	1 μM	Huang et al. (2022)
Co_3_O_4_/CoFe_2_O_4_ hollow nanocubes (HNCs)	Oxidation Reaction	RGB, HSV	Clinical diagnostics, Environmental monitoring	YOLO v3	0.015 μM	Jiang et al. (2023)
CuO/Fe_2_O_3_ heterojunction nanoparticles	Oxidation Reaction	RGB, HSV	Point-of-care (POC) testing, Environmental monitoring	YOLO v3	28 μM, 0.69 μM	Sun M. et al. (2023)
MOF-818	Oxidation Reaction	HSV	Point-of-care (POC) testing	YOLO v3	9.02 μM, 0.05305 μM, 0.8 μM, 0.00076 μM	Yu et al. (2023)
GMP-Cu, ASP-Cu	Oxidation Reaction	CIE L*a*b*	Food safety monitoring	Partial Least Squares Discriminant Analysis (PLS-DA) Linear Discriminant Analysis (LDA), Hierarchical Cluster Analysis (HCA)	5 μM	Yang et al. (2024)
Au NPs@apt	Oxidation Reaction	RGB	Point-of-care (POC) testing, Food safety monitoring	Linear Discriminant Analysis (LDA)	1,000 CFU/mL, 10 CFU/mL	Li et al. (2024)
NH2-MIL-88B (Fe, Ni)	Oxidation Reaction	RGB, HSV	Point-of-care (POC) testing, Food safety monitoring	YOLO v3	0.182 μM, 0.0668 μM	Zhang et al. (2024b)
Bimetallic nickel-cobalt selenides (Ni₀₇₅Co₀₂₅Se)	Oxidation Reaction	RGB	Biomedical diagnostics, Environmental monitoring, Safety screening	Artificial Neural Network (ANN)	5 μM	Lian et al. (2024)
Iron oxide nanoparticles (IONPs)	Oxidation Reaction	RGB	Dental diagnostics	Linear Discriminant Analysis (LDA)	68 CFU/mL	Zhang et al. (2024a)
Au@Ag, Ir@Pd	Oxidation Reaction	RGB	Biosensing, Disease treatment, Environmental management	CatBoost Algorithm	−	Wan et al. (2024)
Fe-N-C, single-atom nanozymes (SANs), Fe-N-C-urea SANs	Oxidation Reaction	RGB	Dental diagnostics	Linear Discriminant Analysis (LDA), Hierarchical Cluster Analysis (HCA)	68 CFU/mL	Zhang L. et al. (2024)

In 2022, (Huang et al., 2022) introduced a deep learning-assisted method employing Fe₃O₄ nanoparticles for real-time visualization and recognition of complex information within latent fingerprints (Huang et al., 2022). The method significantly enhanced the accuracy and depth of fingerprint analysis, demonstrating the powerful impact of combining ML with nanozyme technology in forensic science. The ability to analyze complex patterns with high precision underscores the potential of ML-driven nanozymes for improving forensic diagnostics. Such advancements were mirrored by Mastronardi et al. (2022), who developed a fast colorimetric test to detect cisplatin in biological samples (Mastronardi et al., 2022). Their study demonstrated the capability of ML models to process colorimetric data obtained from a series of reactions, where changes in the intensity of the blue color were directly related to the cisplatin concentration, enabling the precise monitoring of drug levels, as shown in Figure 4B. In another notable advancement, Lu et al., in (2022) developed a metal-nanoparticle-supported nanozyme-based colorimetric sensor array aimed at the precise identification of oral bacteria and proteins (Lu et al., 2022a). Their innovation provided a robust platform for oral health diagnostics, allowing accurate detection of bacterial species and protein markers associated with dental diseases. They highlighted the growing role of nanozyme-based sensors in clinical diagnostics, particularly under conditions requiring rapid and precise detection.

In 2024, (Wan et al., 2024) leveraged ML-accelerated high-throughput computational screening to identify bimetallic nanoparticles with peroxidase-like activity (Wan et al., 2024). Using ML, They significantly streamlined the discovery process, reducing the time and resources required to identify highly active nanozymes. Their study exemplified the synergy between computational techniques and nanotechnology, offering a model for more efficient discovery and development of functional nanozymes. Building on these advancements, (Dang et al., 2022) constructed a Ni-CoMoO_4_ heterostructure with strong Ni–O–Co bonds to enhance multi-functional nanozyme activity (Dang et al., 2022). The heterostructure demonstrated improved catalytic performance, particularly in environmental applications such as pollutant degradation. They highlighted the potential of tailored nanostructures to boost the efficiency and applicability of nanozymes in diverse bioapplications.

To further expand the scope of nanozyme applications, Sun Q. et al. (2023) developed a Mo single-atom nanozyme anchored on a 2D N-doped carbon film (Sun Q. et al., 2023). The system was designed to visually monitor choline levels and evaluate intracellular reactive oxygen species (ROS) generation, thereby providing new insights into the catalytic mechanisms of nanozymes and their roles in cellular processes. They illustrated the dual diagnostic and therapeutic potential of nanozymes, particularly in monitoring and influencing cellular activity. Similarly, Zhang L. et al. (2024) introduced an enhanced “electronic tongue” based on a DNA-encoded nanozyme sensor array for the discrimination and elimination of dental bacteria (Zhang L. et al., 2024). The innovation represented a significant advancement in dental diagnostics, and offered a powerful tool for identifying and targeting pathogenic bacteria in the oral cavity. The integration of DNA technology with nanozyme sensors underscored the interdisciplinary nature of modern biosensing approaches by blending molecular biology with nanotechnology. Yang et al. (2022) provided a comprehensive summary of recent progress in colorimetric sensors based on gold nanoparticles for pathogen detection (Yang et al., 2022).

### 4.2 Integration of nanozymes with ML for advanced bioapplications

The integration of nanozymes with ML represents a transformative approach in advanced bioapplications that combines the unique catalytic properties of nanozymes with the predictive and analytical power of ML algorithms. The synergy has the potential to significantly enhance sensitivity, specificity, and overall performance of biosensing and therapeutic platforms. By leveraging the strengths of both technologies, researchers are focusing on developing innovative solutions that not only improve the detection of critical biomarkers, but also enable more effective treatment and environmental remediation. This section explores recent advancements in the field, highlighting key studies that have demonstrated the potential of combining nanozymes with ML for cutting-edge bioapplications. Table 2 provides a concise overview of various nanozyme-based electrochemical reactions, summarizing their associated electrochemical reactions, detection technologies, applications, ML methods, and limits of detection (LOD). The table highlights the integration of ML techniques for enhancing the performance and specificity of nanozyme-based detection systems across various fields.

**TABLE 2:** Overview of nanozyme-based electrochemical reactions integrated with machine learning for advanced detection technologies and bioapplications.

Nanozyme type	Electrochemical reaction	Detection technology	Application	Machine learning method	LOD	Ref.
MoS2-MWCNTs	Electrocatalytic oxidation of carbendazim (CBZ)	Cyclic voltammetry (CV), Differential pulse voltammetry (DPV)	Detection of CBZ residues in edible agro-products	Artificial Neural Network (ANN)	0.0074 μM	Zhu et al. (2020)
MWCNT-NGr/PEDOT:PSS nanohybrid	Oxidation of amaranth (AM)	Cyclic voltammetry (CV), Differential pulse voltammetry (DPV)	Medical diagnostics, Environmental monitoring, Supercapacitors	Genetic Algorithm-Artificial Neural Network (GA-ANN)	0.015 μM	Xue et al. (2020)
Aptamer-modified C3N4 nanosheets (Apt/C3N4 NSs)	Oxidation of oPD to DAP catalyzed by the Apt/C3N4 NSs	Nanozyme Sensor Array, Ratiometric Fluorescence Detection, Solvent-Mediated Signal Amplification	Non-invasive cancer diagnosis	Linear Discriminant Analysis (LDA), Hierarchical Clustering Analysis (HCA)	2.5 × 10³ particles/mL	Liu X. et al. (2021)
Silver nanoparticles (AgNPs) decorated phosphorene (black phosphorus, BP)	Electrocatalytic oxidation of 8-hydroxy-2′-deoxyguanosine (8-OHdG)	Electrochemical sensing using linear sweep voltammetry (LSV)	Non-invasive medical diagnostics, Monitoring oxidative stress-related diseases	Artificial Neural Network (ANN)	0.2 μM	Sheng et al. (2021)
Amorphous molybdenum sulfide (a-MoSx)	Redox process of baicalin	Cyclic voltammetry (CV), Differential pulse voltammetry (DPV)	Medical diagnostics	Least Squares Support Vector Machine (LSSVM), Artificial Neural Network (ANN)	0.002 μM	Rao et al. (2021)
Graphene-like titanium carbide (Ti2C) Mxene, Au-Ag nanoshuttles (NSs)	Oxidation of carbendazim (CBZ)	SERS detection, Cyclic voltammetry (CV), Differential pulse voltammetry (DPV)	Detection of ultra-trace amounts of carbendazim (CBZ) residues	Artificial Neural Network (ANN), Support Vector Machine (SVM), Relevance Vector Machines (RVM)	0.01 μM	Zhu et al. (2021a)
Flexible 3D porous graphene nanozyme	Oxidation of xanthine and hypoxanthine	Differential pulse voltammetry (DPV)	Detecting the levels of XT and HX, which are indicators of fish spoilage	Artificial Neural Network (ANN)	0.26 μM, 0.18 μM	Zhu et al. (2021b)
Nanocomposite of black phosphorene (BP) with single-walled carbon nanohorns (SWCNH)	Oxidation of 5-hydroxytryptamine (5-HT)	BP-IL-SWCNH modified glassy carbon electrode (GCE)	Monitoring neurotransmitter levels	Artificial Neural Network (ANN)	0.1 μM	Zhu et al. (2021c)
Nanohybrid of phosphorene (BP) and Ti3C2 MXene	Oxidation of α-naphthalene acetic acid (NAA)	Linear Sweep Voltammetry (LSV)	Environmental monitoring	Artificial Neural Network (ANN)	0.0016 μM	Zhu et al. (2021d)
AgNPs/MWCNTs/GO nanohybrid	Oxidation of benomyl (BN)	Differential pulse voltammetry (DPV)	Food safety monitoring	Support Vector Machine (SVM), Least Square Support Vector Machine (LS-SVM)	0.0139 μM	Xu B. et al. (2022)
Carbonized metal–organic framework (C-ZIF-67)	Electrochemical oxidation of NA	Cyclic voltammetry (CV), Square Wave Voltammetry (SWV), Electrochemical Impedance Spectroscopy (EIS)	Electrochemical detection of niclosamide (NA) in agricultural products	Artificial Neural Network (ANN)	0.0003 μM	Lu et al. (2022b)
Cu@Cu2O (CC), Cu@Cu2O@Pd (CCP), Cu@Cu2O@PdAu (CCPA)	Catalytic oxidation of hydrogen peroxide (H2O2)	Sensor array using the synthesized nanozymes (CC, CCP, CCPA)	Cosmetic safety monitoring	k-nearest neighbors (k-NN)	0.982 μM	Chen Y. et al. (2022)
Zn-Co metal-organic framework (MOF), Ti3C2 Mxene, Fe3O4-magnetic graphene oxide (Fe3O4-MGO) nanohybrid	Electrocatalytic oxidation of mycophenolic acid (MPA)	Electrochemical impedance spectroscopy, Voltametric methods	Food safety monitoring	Artificial Neural Network (ANN)	0.021 μM	Ge et al. (2022)
Graphene-like molybdenum selenide (MoSe2-BC)	Oxidation of hesperetin (HP)	Differential pulse voltammetry (DPV)	Food safety monitoring, Environmental management	Least Squares Support Vector Machine (LS-SVM)	0.002 μM	Rao et al. (2022)
Co3O4-CoFe2O4 hollow nanocube	Catalysis of redox reactions	Deep-learning-assisted smartphone biosensing platform	Environmental monitoring	YOLO v3	0.18 μM, 0.015 μM, 8.84 μg mL^−1^	Jiang et al. (2023)
NiCo-MOF, Silver nanoparticles (AgNPs)	Nonenzymatic oxidation of glucose	Cyclic voltammetry (CV)	Assessing the fermentation process and ensuring product quality in liquor brewing	Back-Propagation Artificial Neural Network (BP-ANN)	2.3 μM	Ma et al. (2023)
HNT/BP-AgNPs	Oxidation-Reduction processes	Electrochemical sensor that integrates a screen-printed carbon electrode (SPCE)	Monitoring the safety of food products	Back Propagation Artificial Neural Network with Genetic Algorithm (BP-ANN-GA), Least Squares Support Vector Machine (LS-SVM), Artificial Neural Network (ANN)	0.3 μM	Ge et al. (2023)
Phosphorene nanozyme	Oxidase-like reaction	Differential pulse voltammetry (DPV)	Monitoring drug residues in livestock	Back Propagation Artificial Neural Network with Genetic Algorithm (BP-ANN-GA), Least Squares Support Vector Machine (LS-SVM), Radial Basis Function (RBF), Extreme Learning Machine (ELM)	0.0032 μM	Xiong et al. (2023)
Single-atom nanozymes (SANs), Single-atom catalysts (SACs)	Carbon dioxide electroreduction (CO2 ER)	Density Functional Theory (DFT)	Environmental protection	Ensemble boosting, Random Forest Regression (RFR)	−	Sun and Liu (2024)

In 2021, (Liu M.-X. et al.,) developed a nanozyme sensor array that utilized a sovent-driven approach for enhanced signal amplification in the ultrasensitive detection of exosomal protein, which are crucial biomarkers for cancer identification (Liu M.-X. et al., 2021). Their innovative approach demonstrated exceptional sensitivity and specificity, making it a promising tool for early cancer diagnosis. They underscored the potential of nanozymes for improving the detection of low-abundance biomarkers, which is essential for timely and accurate disease diagnosis. As shown in Figure 4C, Xue et al. (2020) introduced a multi-walled carbon nanotube-N-doped graphene (MWCNT-NGr) nanohybrid integrated with poly (3,4-ethylenedioxythiophene): poly (styrenesulfonate) (PEDOT) for electrochemical applications (Xue et al., 2020). The nanohybrid was specifically designed for intelligent sensors and supercapacitors, and exhibited enhanced electrochemical performance owing to the synergistic effects of the materials. Xue et al. highlighted the potential of combining carbon-based nanomaterials with conducting polymers to improve the efficiency and functionality of electrochemical biosensors and energy storage devices. Similarly, Sheng et al. (2021) introduced a stable nanosilver-decorated phosphorene nanozyme combined with phosphorus-doped porous carbon microspheres (Sheng et al., 2021). Their system was specifically designed for the intelligent sensing of 8-hydroxy-2′-deoxyguanosine, a biomarker associated with oxidative DNA damage often linked to cancer. The integration of nanosilver and phosphorene provided enhanced catalytic activity and stability, marking a significant advancement in cancer detection technology. They illustrated improvements to the performance of biosensors by combining different nanomaterials, particularly in challenging biological environments.

Further, Rao et al. (2021) focused on the green synthesis of an amorphous molybdenum sulfide nanocomposite with biochar microspheres (Rao et al., 2021). The composite was used in a voltammetric sensing platform that exhibited high sensitivity and selectivity for baicalin, a compound with important pharmacological effects. Wang highlighted the potential of using eco-friendly materials to develop advanced biosensors, which are increasingly important for sustainable technology development. Liu Q. et al. (2021) explored the therapeutic applications of nanozymes by developing Au-ZnO-based Trojan nanogenerators activated by ultrasound for targeted electrostimulation and enhanced catalytic therapy for tumors (Ma et al., 2021). Their study presented a novel integration of nanozyme technology with therapeutic applications, offering new avenues for cancer treatment by improving the efficacy of catalytic therapies. They demonstrated the versatility of nanozymes not only as diagnostic tools, but also as active agents in therapeutic interventions.

In 2022, (Xu B. et al., 2022) developed a Ni-CoMoO4 heterostructure featuring robust Ni–O–Co bonds to enhance multi-functional nanozyme activity (Xu B. et al., 2022). The heterostructure exhibited enhanced catalytic performance, particularly in environmental applications such as pollutant degradation. Chen et al. showcased the potential of nanozymes in addressing environmental challenges and highlighted their role in environmental remediation. Finally, (Lu X. et al., 2022) explored the synergy between ML and nanozyme technology by developing a ML strategy to optimize the performance of electrochemical sensors and supercapacitors using carbonized metal-organic frameworks (MOFs) (Lu X. et al., 2022). The application of ML algorithms significantly improved the sensitivity and accuracy of the sensors, demonstrating the powerful role that ML could play in refining and enhancing nanozyme-based systems. They indicated the growing trend toward integrating computational approaches with nanotechnology to achieve better performance and more precise control over sensor characteristics.

## 5 Challenges and future perspectives

Recent advancements in nanozyme-based detection technologies, particularly the integration of ML, have shown great promise for clinical and environmental applications. However, several challenges must be addressed to fully realize their potential. One of the primary challenges is to ensure the quality and robustness of the data used in ML models. The success of ML-driven diagnostic tools depends heavily on the diversity, accuracy, and relevance of training data (Park et al., 2023; Leem et al., 2022). Comprehensive datasets that accurately represent real-world conditions are crucial for effective generalization of ML models across different scenarios. Without high-quality data, these models may produce inconsistent or inaccurate predictions, limiting their reliability in clinical diagnostics or environmental monitoring (Yoon et al., 2024a; Jeon et al., 2022a). Therefore, the development of extensive, high-quality datasets is essential for the advancement of ML-integrated nanozyme technology.

Another significant challenge involves scaling up the production of nanozyme-based technologies, while maintaining consistent quality and performance (Ai et al., 2022; Singh et al., 2023). Transitioning from laboratory-scale synthesis to commercial production presents difficulties in ensuring that each nanozyme system meets the stringent quality standards. Additionally, incorporating sophisticated ML algorithms into these technologies in a cost-effective manner is crucial for their widespread adoption (Lowe et al., 2022; Zeebaree, 2024). Overcoming the challenges related to scalability and cost-effectiveness is critical for the successful commercialization of nanozyme-based technologies. Furthermore, the lack of standardized protocols for the synthesis, testing, and validation of nanozyme-based detection methods poses a challenge to their broader adoption. Establishing standardized methods is vital to ensure reproducibility across studies and applications (Jeon H.-J. et al., 2022; Jeon et al., 2021; Park et al., 2021). Standardization would also facilitate comparisons between research groups and streamline the regulatory approval process, which is necessary for the commercial deployment of these technologies.

Looking towards the future, specific areas of research should focus on improving data quality, scalability, and standardization in nanozyme technologies. The integration of ML with nanozyme technology offers immense potential to enhance the accuracy, efficiency, and applicability of both colorimetric and electrochemical detection systems across various bioapplications (Qian et al., 2022). In the context of colorimetric detection, advanced image-processing techniques, such as color correction, normalization, and transformation are necessary to standardize and improve the accuracy of detection across different devices. ML, with its ability to analyze complex datasets, can significantly enhance the precision and reliability of these systems by learning from data and adapting to various conditions.

Similarly, in the field of electrochemical detection, ML can optimize the interpretation of complex electrochemical signals, thereby improving the sensitivity and specificity of these methods. Techniques such as cyclic voltammetry and differential pulse voltammetry, when integrated with ML algorithms like artificial neural networks (ANNs) and support vector machines (SVMs), can be fine-tuned to detect trace amounts of analytes with higher accuracy (Kurani et al., 2023; Ragab et al., 2019). The approach can be particularly useful in applications such as environmental monitoring and non-invasive medical diagnostics, where detecting low concentrations of substances is critical. Furthermore, the potential of optical hyperspectral imaging (HSI) to obtain more detailed spectral information beyond the primary RGB colors represents a promising avenue for improving both colorimetric detection methods (Jeon et al., 2022b; Yoon et al., 2024b). Recent advancements have made it possible to implement low-cost hyperspectral imaging techniques on smartphones, thereby enhancing the accuracy of glucose detection and other bioapplication analyses.

In summary, future research should focus on addressing key challenges, including data quality, scalability, and standardization, while exploring new ML-driven advancements in detection technologies. By overcoming these challenges, the field can move towards the development of powerful, precise, and accessible diagnostic tools that have wide-ranging clinical and environmental applications. The continued integration of nanozymes with machine learning will likely drive further innovations, leading to more precise, reliable, and widely available detection technologies that can be applied across a range of clinical and environmental settings.
